# Unraveling the importance of functionally extreme tadpole types to functional diversity: a case study in temperate montane streams

**DOI:** 10.1186/s12983-023-00485-0

**Published:** 2023-02-06

**Authors:** Jing Lan, Zijian Sun, Jianyi Feng, Chunlin Zhao, Da Kang, Wenbo Zhu, Tian Zhao, Shengqi Su

**Affiliations:** 1 College of Fisheries, Southwest University, Chongqing, 400715 China; 2 CAS Key Laboratory of Mountain Ecological Restoration and Bioresource Utilization and Ecological Restoration Biodiversity Conservation Key Laboratory of Sichuan Province, Chengdu Institute of Biology, Chinese Academy of Sciences, Chengdu, 610041 China

**Keywords:** Amphibian, Functional vulnerability, Ecomorphological traits, Functionally extreme species, Microhabitat variables

## Abstract

**Background:**

Functional diversity is important to maintain ecosystem functioning. Species with different ecomorphological traits may display distinct functional roles in ecosystems. Accordingly, functionally extreme species are more important as they can exhibit specific strategies. However, little is known about the distribution patterns of functionally extreme species at a local scale and whether the prior extinction of extreme species can cause significant effects on functional diversity. In addition, no empirical studies have been conducted on the microhabitat determinants of extreme species to maintain the functional diversity.

**Results:**

This study collected 1470 tadpoles belonging to 6 families and 20 anuran species. These species were subsequently divided into 65 functional entities based on their developmental stages to incorporate intraspecific traits variability. As a result, we detected seven extreme functional entities, accounting for 10.7% of the total number of entities. Moreover, the prior extinction of extreme entities can lead to a significant decrease in functional diversity compared with the random extinction of entities. Microhabitat variables such as conductivity, water depth, and current velocity determined the distribution of extreme entities.

**Conclusion:**

Although the functionally extreme entities only represented a small proportion of the total number of tadpoles, they played irreplaceable roles in maintaining functional diversity. Their extinction may induce high functional vulnerability in tadpole communities. Therefore, anuran species with extreme tadpole traits need to be projected for amphibian conservation.

**Supplementary Information:**

The online version contains supplementary material available at 10.1186/s12983-023-00485-0.

## Background

Biodiversity has a positive relationship with ecosystem functioning and has attracted the attention of ecologists for a long time [1, 2]. However, most previous studies only focused on the taxonomic facet of biodiversity (e.g., species richness), despite biodiversity contains multidimensional information on functional and phylogenetic facets [3, 4]. In recent decades, with the development of functional ecology, increasingly studies have started to quantify the functional dimensions of biodiversity (i.e., functional diversity) using the functional characteristics of species in a particular region [5, 6]. As a result, functional diversity is considered to be more sensitive and predictive in quantifying the relationship between biodiversity and ecosystem functioning [7]. For instance, the increased fish functional diversity can promote material circulation and energy flow in a tropical stream in Água Azeda Creek, Bahia, Brazil [8]. On the other hand, for birds in some northern hemisphere countries such as China, Russia, and Finland, the decrease in their functional diversity strongly threatens the ecosystem functioning of forests due to human activity and seed dispersal [9].

Among all the organisms, amphibians have rich diversity and contribute to many key ecosystem processes, such as promoting material exchange, regulating nutrient cycling and energy flow, and controlling pests [10]. This is especially true for their larval tadpoles, which play critical functional roles in freshwater ecosystems, especially in montane streams [11]. For instance, tadpole excretion and digestion behaviors can promote nutrient circulation and litter decomposition in streams [12]. Therefore, their consumption activities and interactions can strongly affect stream food web structures [13]. Previous tadpole functional studies usually selected some ecomorphological traits as their functional traits [14, 15]. Although these traits do not reflect the fundamental functions of tadpoles in ecosystems, they can also reflect critical ecological functions through food acquisition and locomotion to regulate tadpole contributions to ecosystem processes [16]. Accordingly, studies have been conducted to investigate the responses of tadpole functional traits to perturbations [17] and to explore the spatial patterns of tadpole functional diversity [18–20]. However, little attention has been paid to the distinct ecological strategies of different tadpoles.

The distribution patterns of traits of organisms reflect their distinct ecological strategies [21]. As the trait distribution is often unimodal in animals [22], species with the core distribution are generalists, while species distributed in tails can be considered as the specialists [23]. Although only a few species had extreme traits in communities [24], the importance of functionally extreme species to functional diversity is still unclear. Moreover, it is widely recognized that species traits are strongly affected by environmental conditions (e.g., plants: [25], fish: [26], and amphibian tadpoles: [5]). However, empirical evidence related to the environmental determinants of the distribution of extreme species is still limited.

**Fig. 1:**
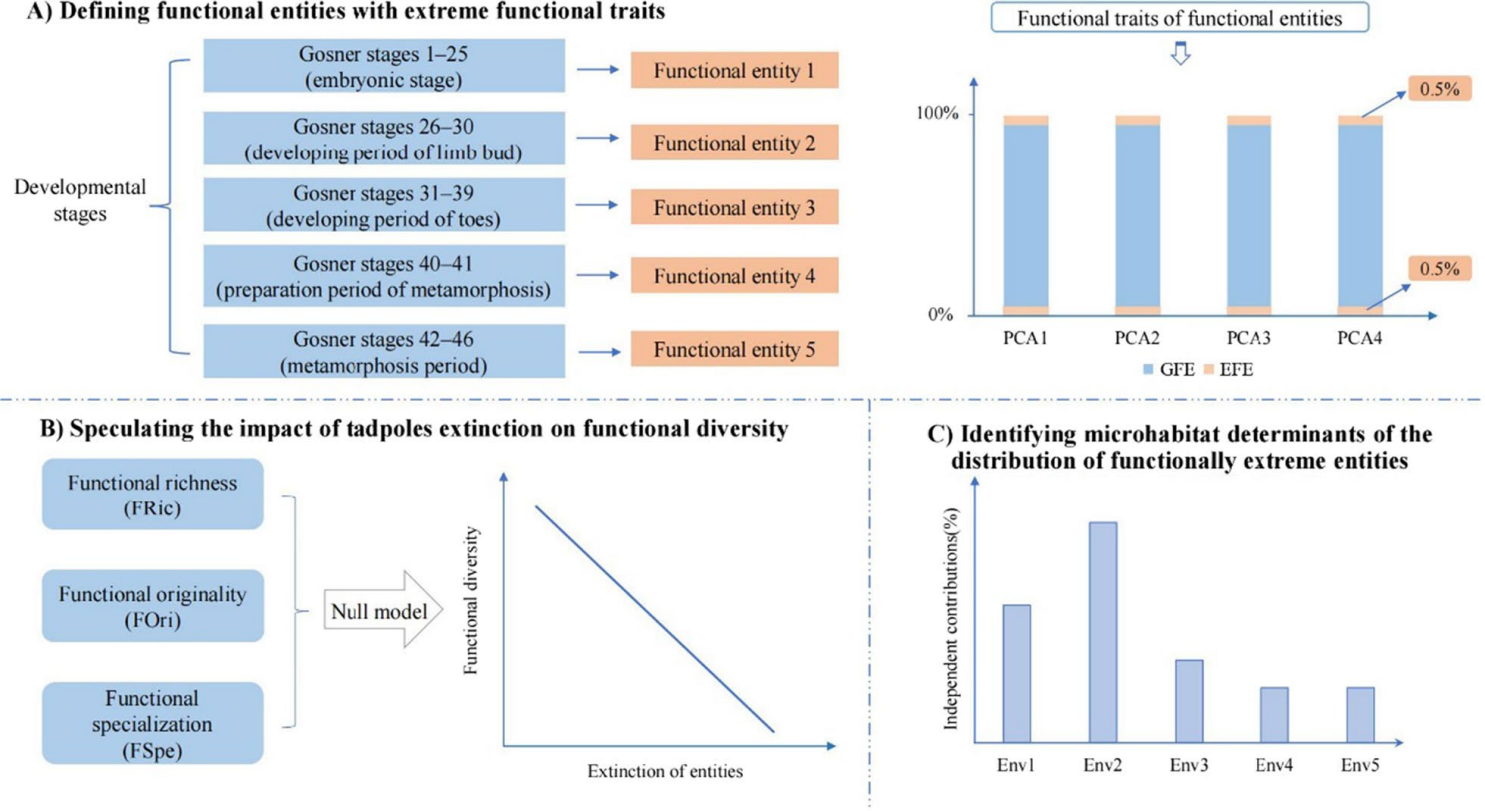
General framework of the present study. **A** Defining functional entities with extreme functional traits. **B** Speculating the impact of tadpoles extinction on functional diversity. **C** Identifying microhabitat determinants of the distribution of extreme functional entities

In the present study, we used anuran tadpoles in montane streams of a temperate forest as models to explore the importance of functionally extreme species to functional diversity. Specifically, we aim to (1) identify the extreme functional tadpole types in the studied montane streams, (2) investigate whether the prior extinction of extreme tadpole types can cause significant effects on functional diversity indices than the random extinction of tadpoles, and (3) quantify the microhabitat determinants of the distribution of extreme tadpole types (Fig. 1).

## Materials and methods

### Study area

This study was conducted in the montane streams of Mount Emei (29°16′–29°43′ N, 103°11′–103°37′ E) located on the southwestern edge of the Sichuan Basin, China. The highest altitude of this mountain is 3099 m, and the altitude span is about 2600 m [27]. This area belongs to a typical subtropical climate. However, a large elevational gradient induced four distinct climate zones from low to high elevations. Specifically, it is the subtropical climate below 1500 m, which was covered by evergreen broad-leaved forest. The warm temperate climate is between 1500 and 2100 m, and the main vegetation cover is evergreen, deciduous broad-leaved mixed forest. Middle temperate climate can be detected between 2100 and 2500 m, with broad-leaved and coniferous forests dominating this area. In addition, it is the sub-cold zone climate with cold temperate coniferous forest above 2500 m [28]. Abundant vegetation and freshwater resources provide various microhabitats for amphibians, and 35 amphibian species were recorded in this area based on literatures [29].

### Field-work

Field-work was conducted in 18 streams, covering an elevational gradient from 485 to 2865 m (Additional file 1: Table S1). These streams contained diverse microhabitats, providing suitable habitats for different tadpoles. Only 300 m of each stream was selected and sampled, as high waterfalls or cliffs can usually block the streams. Specifically, tadpoles in each stream were sampled between 20:30 h and 23:00 h at night using dip nets (opening diameter: 40 cm; depth: 35 cm; mesh size: 3 mm; RBF326, Renniaofei, China) from May to June in 2018 and 2019, respectively, with one stream being sampled per night. The developmental stages of sampled tadpoles were determined according to Gosner [30]. For individuals that can be directly identified to species by morphological characteristics (e.g., *Leptobrachium boringii* and *Quasipaa boulengeri*), we collected at least 15 individuals of each species at the different developmental stages and brought them back to the laboratory for further analyses. The rest individuals were counted and released back to their original habitats. For individuals that cannot be identified to species directly by external morphology (e.g., *Megophrys omeimontis* and *Megophrys shapingehsis*), all the sampled individuals were brought back to the laboratory and identified to species using DNA barcoding analyses based on mitochondrial 16S rRNA gene fragments [5].

We also recorded 12 microhabitat variables based on previous studies showing that they can potentially determine tadpole distribution and diversity [5, 31, 32]. These variables included conductivity (Con), water depth (Wd), river width (Rw), dissolved oxygen (Do), water pH (pH), water temperature (Wt), total nitrogen (TN), total phosphorus (TP), current velocity (Cv), ammonia nitrogen (NH_4_-N), chlorophyll *a* (Chl. *a*), and substrate type (Sub). In addition, a previous study has provided details of the measurement approaches [5].

### Functional traits and functional entities

Based on previous studies [15, 33], we selected 11 functional traits related to the two key ecological functions (i.e., food acquisition and locomotion) of tadpoles displayed in aquatic ecosystems. Specifically, there are four functional traits related to food acquisition (i.e., oral disk shape, oral disk position, eye position, and gut length), and six functional traits are related to locomotion (i.e., tail shape, tail position, tail throttling, trunk bending shape, spiracle position, and body section shape). In addition, mass is related to both food acquisition and locomotion. These traits were calculated as the ratios of 11 morphological traits (Additional file 1: Table S2). Details of the measurement of these traits can be found in a previous report [5].

In the past two decades, increasing studies have reported the importance of intraspecific traits variability in functional ecological studies [34–36], and they suggested that species should be divided into different functional entities according to their life stages (e.g., [6, 37]). Therefore, individuals within tadpole of each anuran species were divided into five functional entities based on their developmental stages following the description in a previous study [38]. These stages included functional entity 1 (Gosner stages 1–25; embryonic stage), functional entity 2 (Gosner stages 26–30; developing period of limb bud), functional entity 3 (Gosner stages 31–39; developing period of toes), functional entity 4 (Gosner stages 40–41; preparation period of metamorphosis), and functional entity 5 (Gosner stages 42–46; metamorphosis period [5]).

### Statistical analyses

First, we conducted a principal component analysis (PCA) based on scaled 11 functional traits of all tadpole functional entities. The first four principal components had their eigenvalues > 1, which were selected to construct the functional traits space. They occupied 76.74% of the initial inertia in trait values (PC1 = 27.25%, PC2 = 23.29%, PC3 = 15.74%, and PC4 = 10.46%, respectively). Following Su et al. [24], the 0.5% of entities with the lowest values and the 0.5% of entities with the highest values on each of the four PCA axes were defined as the extreme functional entities (EFE), and the remaining were defined as the general functional entities (GFE).

All the entities were gathered together as the community (i.e., the regional pool), as amphibians could move freely in the mountain. The functional diversity of the community was assessed based on three indices, including functional richness (FRic), functional originality (FOri), and functional specialization (FSpe). We selected these indices because their calculations involved in extreme functional entities [39]. Specifically, functional richness indicated the volume of functional space occupied by all entities [7]. Functional originality referred to a metric of original isolation when entities occupied functional space in a community, quantifying how altered functional redundancy between entities [39, 40]. Functional specialization reflected the specialization of the entities in the functional space, showing how the abundance of extreme entities increased [24, 39, 41, 42]. We generated null models to explore whether the prior extinction of EFE/GFE can cause significant effects on functional diversity indices than the random extinction of entities from the regional pool. Finally, we designed an appropriate randomization procedure to test the changes in functional diversity indices with the extinct process of EFE/GFE (see Additional file 1: Appendix Figure S1 for details). The null models randomized the tadpole assemblages after EFE/GFE had been extinct. The randomization process was carried out 999 times for each index, and the *P* values were calculated to indicate the statistical significance. In addition, standardized effect sizes (SES) were calculated as the differences between the observed functional diversity values and the random functional diversity values standardized by the standard deviation.

Finally, we used generalized linear models (GLMs) to explore the microhabitat determinants of the richness and the relative abundance of EFE, respectively. Specifically, we used a Poisson distribution with a logarithmic link function in the models, and different models were compared by deleting variables one by one from the global model. Because of the small sample size, the best-fitted models were selected based on the AICc values [43]. Moreover, model averaging was performed if needed [44]. Furthermore, the significant variables were identified when *P* < 0.05. After that, hierarchical partitioning analyses were conducted to quantify the relative contribution of each microhabitat variable in the best model to the richness and to the relative abundance of EFE, respectively.

All calculations and statistical analyses were performed in R 4.0.3 [45]. PCA analyses were conducted using *vegan* package [46]. GLMs were performed using the *lme4* package [47]. Hierarchical partitioning was performed using *hier.part* package [48]. The null models were performed by *picante* package [49].

## Results

### Functional entities with extreme traits

Within two years of sampling, we collected 1496 tadpole individuals, which belonged to 20 anuran species from 6 families (Table 1). These individuals were subsequently divided into 65 functional entities based on their developmental stages, with each functional entity being measured for 23.0 ± 47.2 (mean ± SD) individuals. Specifically, the common entities were tadpole of *Leptobrachium boringii* 1, *Oreolalax omeimontis* 1, and *Megophrys omeimontis* 3, accounting for 20.7%, 12.4%, and 9.5% of the total number of individuals, respectively. The rare entities were tadpole of *Oreolalax major* 1, *Leptobrachella oshanensis* 4, and *Fejervarya multistriata* 5, accounting for 0.06%, 0.12%, and 0.17% of the total number of individuals, respectively.

**Table 1:** List of tadpole of anuran species detected in Emei Mount

Family	Genus	Species	Abbreviation	Number of functional entities
*Bufonidae*	*Bufo*	*Bufo gargarizans*	bug	5
*Dicroglossidae*	*Fejervarya*	*Fejervarya multistriata*	fem	4
	*Quasipaa*	*Quasipaa boulengeri*	qub	5
*Megophryidae*	*Leptobrachium*	*Leptobrachium boringii*	leb	4
	*Megophrys*	*Megophrys minor*	mem	4
	*Megophrys*	*Megophrys omeimontis*	meo	5
	*Megophrys*	*Megophrys shapingensis*	mes	1
	*Oreolalax*	*Oreolalax major*	orm	1
	*Oreolalax*	*Oreolalax omeimontis*	oro	5
	*Oreolalax*	*Oreolalax popei*	orp	3
	*Oreolalax*	*Oreolalax schmidti*	ors	2
	*Paramegophrys*	*Paramegophrys oshanensis*	pao	4
*Microhylidae*	*Microhyla*	*Microhyla fissipes*	mif	1
*Ranidae*	*Amolops*	*Amolops chunganensis*	amc	1
	*Amolops*	*Amolops granulosus*	amg	1
	*Odorrana*	*Odorrana margaretae*	odm	5
	*Pelophylax*	*Pelophylax nigromaculatus*	pen	5
	*Rana*	*Rana omeimontis*	rao	4
*Rhacophoridae*	*Rhacophorus*	*Rhacophorus dugritei*	rhd	1
	*Rhacophorus*	*Rhacophorus omeimontis*	rho	4

**Fig. 2:**
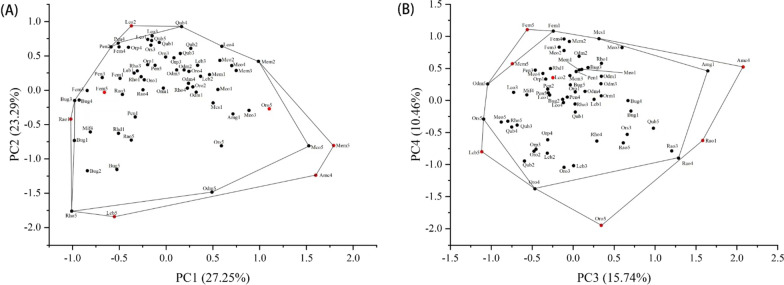
The distribution of tadpole functional entities in the functional space. **A** Functional space constructed by the first two PC axes. **B** Functional space constructed by PC3 and PC4. The outer convex polygon represents the area occupied by all functional entities in the functional space, while the inner convex polygon represents the area occupied by the GFE in the functional space. The red dots represent EFE, and the black dots represent GFE. Details of the abbreviations are in Table 1

In the functional space, PC1 was mainly contributed by functional traits related to locomotion, with higher values indicating stronger mobility. PC2 was mainly contributed by functional traits related to food acquisition, and the higher values in PC2 demonstrated that tadpoles preferred to prey on the surface of the water. Moreover, these tadpoles also exhibited strong detection but weak digestion ability. In addition, PC3 and PC4 were mainly driven by spiracle position and mass, respectively. Based on the distribution in the four PC axes, seven functional entities were identified as EFE, accounting for 10.7% of the total number of functional entities. Specifically, these entities were tadpole of *L. boringii* 5, *Amolops chunganensis* 4, *F. multistriata* 5, *Megophrys minor* 5, *O. omeimontis* 5, *L. oshanensis* 2, and *Rana omeimontis* 1 (Fig. 2). Furthermore, they filled 9.75%, 3.29%, 14.25%, and 19.38% of the range of PC1, PC2, PC3, and PC4, respectively (Additional file 1: Figure S2).

### Effects of tadpole extinction on functional diversity

**Fig. 3:**
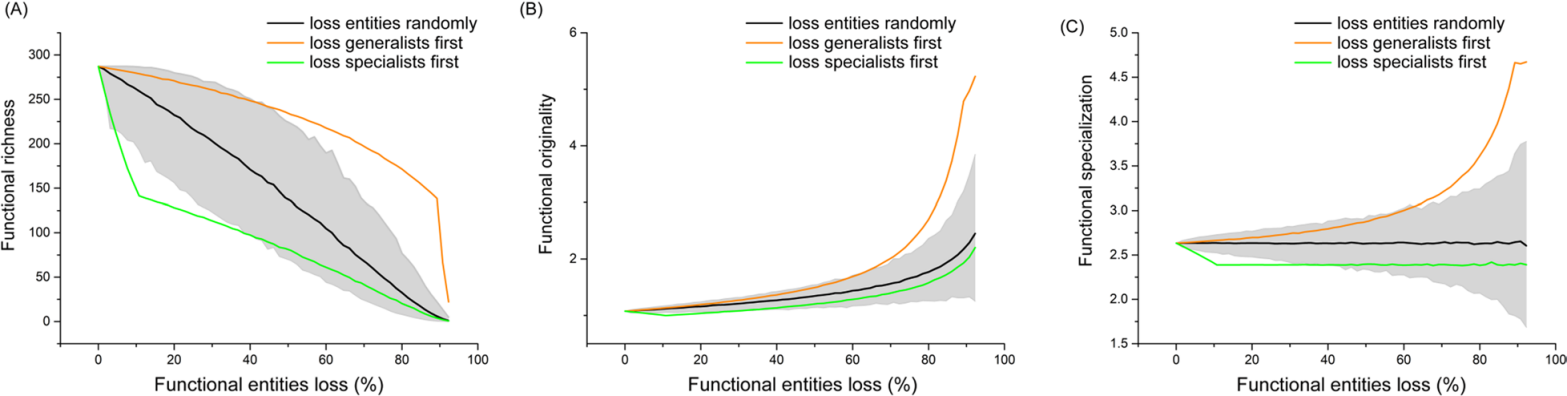
The effects of entities extinction on functional richness (**A**), functional originality (**B**), and functional specialization (**C**). The green solid line is the change of the prior extinction of EFE. The orange solid line is the change of the prior extinction of GFE. The black solid line is the change of the random extinction of entities. And the gray area represents the 95% confidence interval

Overall, the prior extinction of EFE had significant effects on functional diversity compared to those calculated from the random extinction of functional entities (Fig. 3). Specifically, functional richness could significantly decrease after prior extinction of one EFE (SES = − 2.087, *P* = 0.028). However, there was no significant effects on functional specialization (SES = − 1.624, *P* = 0.81) and functional originality (SES = − 1.497, *P* = 0.058). All the functional diversity indices for the prior extinction of two to seven EFE were significantly lower than those calculated based on random extinction (Additional file 1: Table S4; Fig. 3). In contrast, the prior extinction of one to seven GFE had no significant effects on all the functional diversity indices (Additional file 1: Table S4; Fig. 3).

### Environmental determinants of the distribution of EFE

**Fig. 4:**
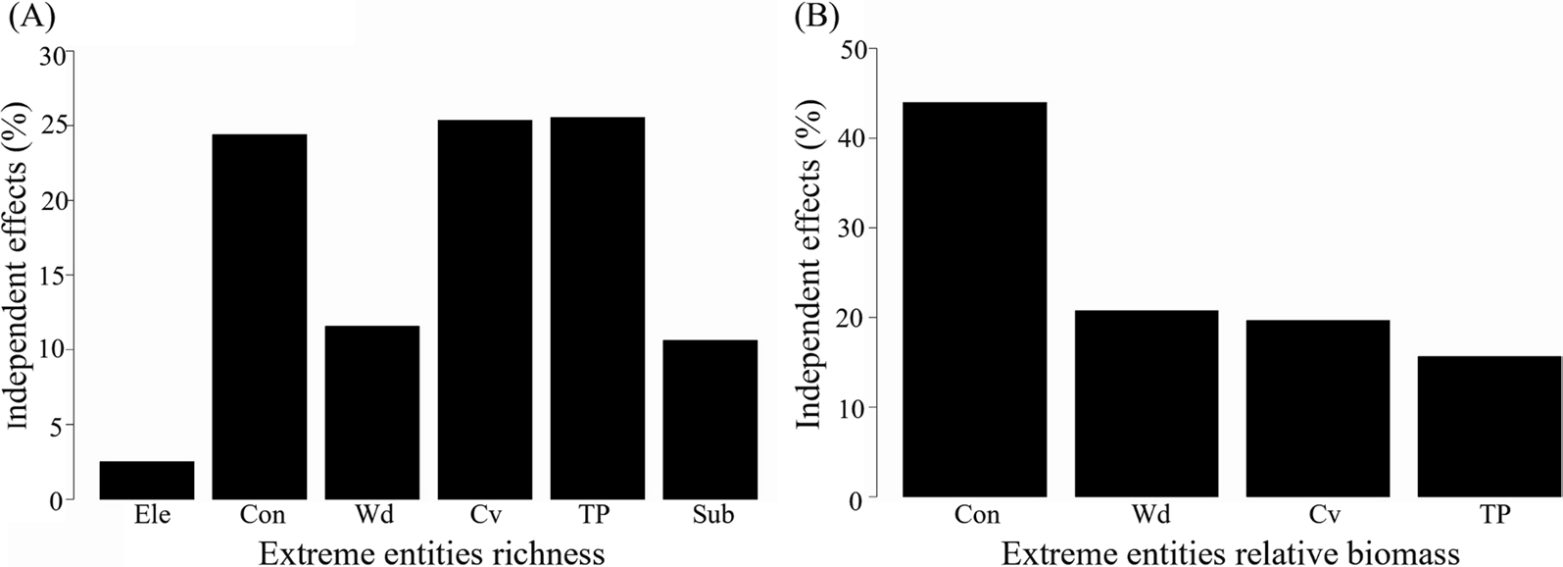
Results of hierarchical partitioning showing the independent contribution of selected microhabitat determinants to the variation of richness of functionally extreme tadpole types (**A**), and relative biomass (**B**). Details of abbreviations are in Table 2

In the best model constructed by GLMs, six microhabitat factors (i.e., elevation, conductivity, water depth, current velocity, total phosphorus, and substrate type) significantly impacted the distribution of EFE richness (Table 2 and Additional file 1: Table S3). Specifically, conductivity and substrate type were significantly and positively correlated with EFE richness (*P* < 0.05), while water depth, elevation, current velocity, and total phosphorus had negative and significant correlations with EFE richness (*P* < 0.05). The results of hierarchical partitioning showed that total phosphorus (25.5%), current velocity (25.3%), and conductivity (24.4%) were the three most important factors contributing to the variance of EFE richness. Moreover, water depth and substrate type contributed 11.6% and 10.6% to the variance of EFE richness, respectively. In addition, the independent contribution of elevation was only 2.5% (Fig. 4A).

**Table 2:** The best model selected by AICc values of the GLMs for the distribution of EFE richness

	Deviation	Standard Error	*t* value	*P* value
(Intercept)	11.310	2.952	3.832	**< 0.001**
Ele	− 0.004	0.002	− 2.471	**0.013**
Con	0.012	0.003	4.180	**< 0.001**
Wd	− 0.123	0.027	− 4.500	**< 0.001**
Cv	− 13.580	3.050	− 4.451	**< 0.001**
TP	− 383.300	78.170	− 4.903	**< 0.001**
Sub	0.405	0.133	3.045	**0.002**

Significant *P* values are in bold. Details of the abbreviations are as follows: Ele, Elevation; Con, conductivity; Wd, water depth; Cv, current velocity; TP, total phosphorus; Sub, substrate type

In terms of the distribution of the relative biomass of EFE, there were four significant microhabitat factors (i.e., conductivity, water depth, current velocity, and total phosphorus; Table 3 and Additional file 1: Table S3) in the best model constructed by GLMs. Specifically, conductivity had a significant and positive correlation with the relative biomass of EFE (*P* < 0.05), while water depth, total phosphorus, and current velocity were significantly and negatively correlated with it (*P* < 0.05). Hierarchical partitioning analyses indicated that the independent contribution of conductivity to the variance of EFE relative biomass was 44.0%, followed by water depth (20.7%), current velocity (19.7%), and total phosphorus (15.6%; Fig. 4B).

**Table 3:** The best model selected by AICc values of the GLMs for the distribution of the relative biomass of EFE

	Deviation	Standard Error	*t* value	*P* value
(Intercept)	0.221	0.088	2.506	**0.017**
Con	0.001	0.000	3.348	**0.002**
Wd	− 0.005	0.002	− 2.736	**0.009**
Cv	− 0.262	0.116	− 2.255	**0.030**
TP	− 6.122	2.306	− 2.655	**0.012**

Significant *P* values are in bold. Details of the abbreviations are in Table 2

## Discussion

The present study revealed that seven of the 65 anuran tadpole functional entities we sampled were identified as EFE. Although these EFEs accounted for a small proportion of the total number of tadpole entities, they had an important contribution to each PC axis. Interestingly, these functional entities were either in the early developmental stage (e.g., *R. omeimontis* 1 and *L. oshanensis* 2) or in the metamorphosis period (e.g., *L. boringii* 5, *F. multistriata* 5, *M. minor* 5, and *O. omeimontis* 5), exhibiting distinct eco-morphology from other entities. Specifically, *O. omeimontis* 5, *M. minor* 5, and *A. chunganensis* 4 were distributed in the functional space with positive values for PC1, but negative values for PC2. Our results were compensated with previous findings showing that the locomotion of these tadpoles was high acceleration and/or maneuverability and low magnitude of vertebral curvature [41, 50]. This is probably because they were close to completing the metamorphosis and thus exhibited more vital locomotion ability. These tadpoles also had higher gut length, lower oral disk shape, and eye position values, indicating that the digestion ability was poor, and their prey detection was more focused on the water surface. This is because these tadpoles were in the metamorphosis period, and their functional traits exhibited significant changes to adapt living from water to land [51]. Moreover, *F. multistriata* 5, *R. omeimontis* 1, and *L. boringii* 5 were distributed in the functional space with negative values for PC1 and PC2. Accordingly, the locomotion of these tadpoles was low acceleration and/or high magnitude of vertebral curvature [15, 50, 52]. This may be because these tadpoles either had poor locomotion ability (i.e., *R. omeimontis* 1) or relied more on the limbs to move (i.e., *F. multistriata* 5 and *L. boringii* 5). In addition, *L. oshanensis* 2 was distributed in the functional space with negative values for PC1, but positive values for PC2. Therefore, these tadpoles relied more on the vertebral column to swim, and they can detect prey easily on both sides of the body. Consequently, they can swim and prey for food agilely [53].

The results of the null models indicated that the prior extinction of EFE can cause a significant decrease in tadpole functional richness. These results showed that despite EFE was rare in a community, they occupied most of the functional space. Therefore, our results were consistent with previous studies showing that the functional richness of a community was mainly supported by EFE [39, 42]. Moreover, the extinction of EFE in the community can cause some vacant functional space, leading ecological resources not to be effectively utilized in the ecosystems [54]. Indeed, montane streams were fragile ecosystems, and their energy input mainly relied on leaf litter decomposition [55]. The vacancy of ecological space caused by the extinction of EFE would break the ecological balance and thus decrease the stability of ecosystems. More importantly, the prior extinction of tadpole EFE can also induce a significant decrease in functional specialization and functional originality, causing an increase in functional redundancy and functional vulnerability in the community [39]. Consequently, these communities can become less resistant and resilient when facing human disturbances [39, 56]. The increase in functional redundancy is also related to the weak and asymptotic relationship between species richness and ecosystem functioning because of the increased functional homogenization [56, 57]. Furthermore, high homogenization can reduce spatial diversity and potentially cut down the ecosystem functioning, thus jeopardizing ecosystem services [58].

The results of GLMs showed that EFE tended to live in water conditions with high conductivity, shallow water, low current velocity, and low phosphorus. These environments are usually related to the high abundance of food resources [52, 59, 60], thus can provide suitable habitats for their growth (e.g., *R. omeimontis* 1 and *L. oshanensis* 2). Previous studies indicated that the adults of *R. omeimontis* and *L. oshanensis* preferred to spawn in still and clean water bodies [53]. Therefore, the selection of adult breeding sites may also determine the distribution of tadpole EFE. Moreover, these water conditions can also benefit the growth of aquatic plants, associated with high habitat heterogeneity [61]. Other EFE, such as *F. multistriata* 5, *L. boringii* 5, *M. minor* 5, and *O. omeimontis* 5 prefer to live in these places as they can find abundant shelters during metamorphosis [53].

Overall, this study identified the anuran species with extreme tadpole traits in streams of Mount Emei. These species exhibited specific ecomorphological traits and occupied most of the functional space. More importantly, although EFE accounted for only a small proportion (10.7%) of the total number of entities, they played irreplaceable roles in maintaining the functional diversity of the community. Accordingly, EFE should be better concerned and protected to preserve tadpole diversity. Furthermore, since EFE reflects the functional vulnerability of ecosystems, future research can explore how the extinction of EFE will affect ecosystem functioning. In addition, since the abundance of EFE was not considered in the present study, functional diversity indices incorporating entity traits and abundance could be applied to better understand the effects of EFE on biodiversity. In addition, the EFE distribution was strongly determined by microhabitat variables such as conductivity, water depth, and current velocity. Therefore, microhabitat protection should be the priority to maintain the richness and abundance of EFE.

## Supplementary Information

**Additional file 1**: Supplementary material.

## Data Availability

Data are available from the authors upon reasonable request.
