# Supplementary material for: Unraveling the importance of functionally extreme tadpole types to functional diversity: a case study in temperate montane streams
Source: Front Zool. 2023 Feb 6;20:7. doi: 10.1186/s12983-023-00485-0 (PMC9900998; doi:10.1186/s12983-023-00485-0)
Supplement: Supplementary file 1 — Additional file 1: Supplementary material. [file 12983_2023_485_MOESM1_ESM.docx]

**Supplementary material**

**
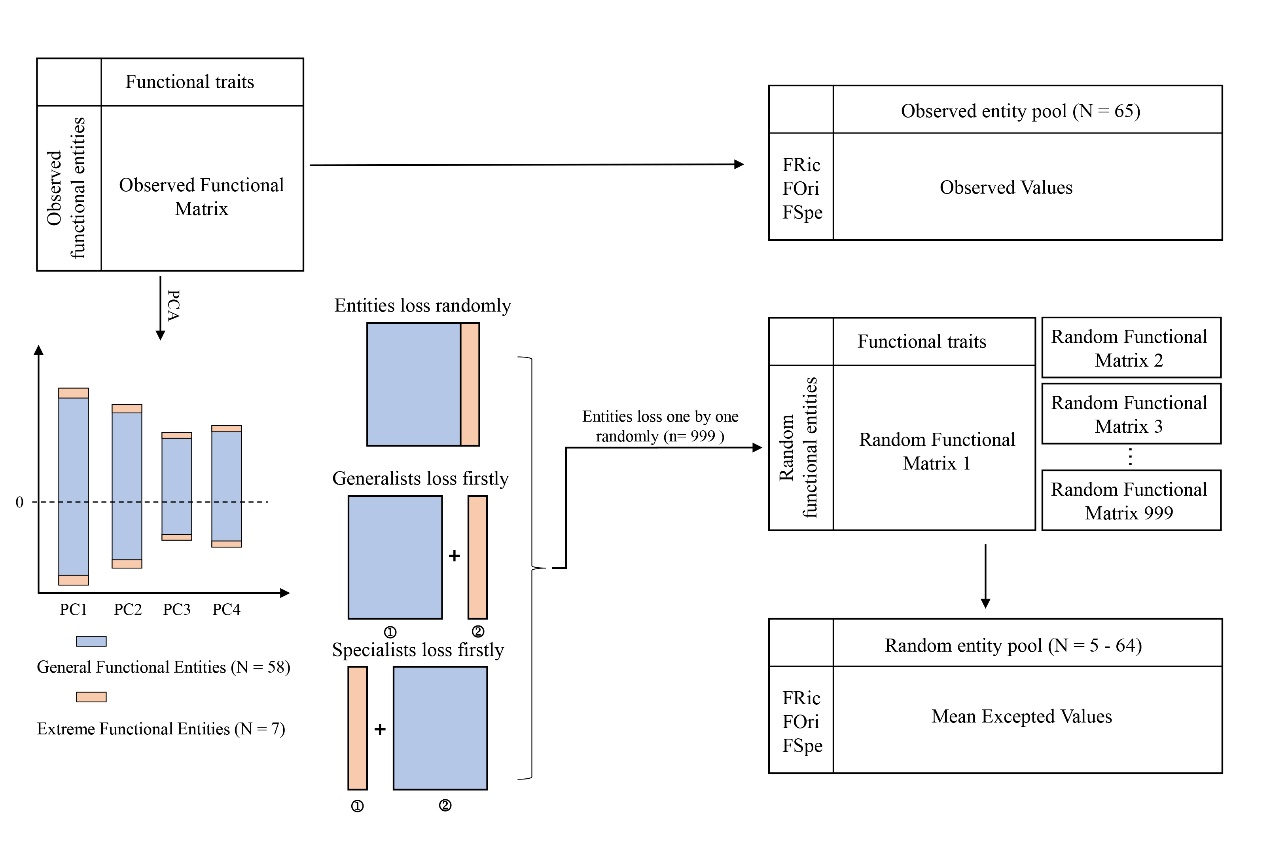
**

**Figure S1** The extinct process of EFE/GFE from the regional pool by null models.

**
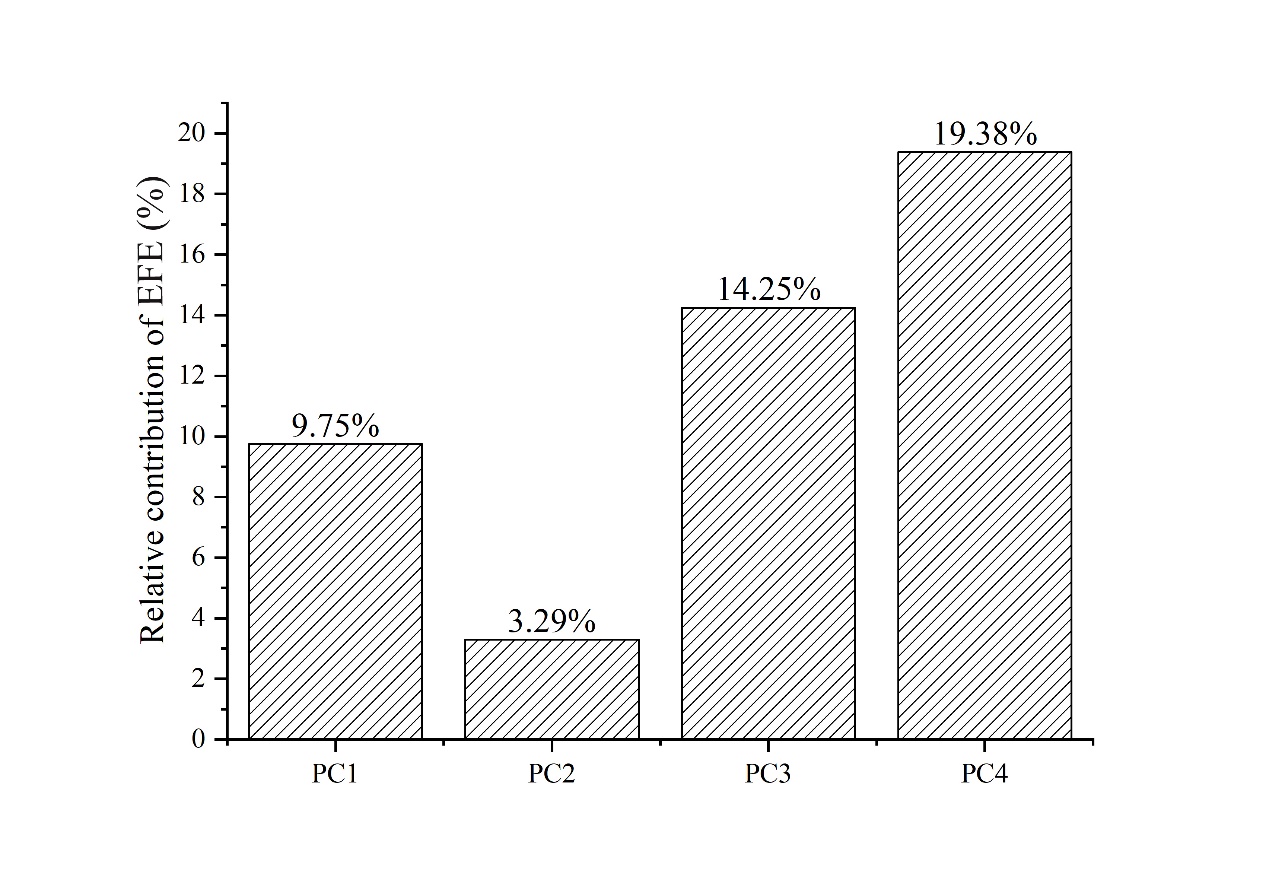
**

**Figure S2** Relative contribution of EFE to the first four PCA axes.

**Table S1** Geographic locations of transects sampled in Emei Mount.

| Transects | Longitude（E） | | Latitude（N） | Elevation（m） |
| --- | --- | --- | --- | --- |
| Huangwanfangqu | | 103.4367 | 29.5824 | 485 |
| Huangwan | | 103.4377 | 29.5874 | 509 |
| Baoguosi | | 103.4408 | 29.5689 | 563 |
| Shenshuigedahe | | 109.4039 | 31.4407 | 638 |
| Lianghekou | | 103.4106 | 29.5866 | 658 |
| Qingyinge | | 103.3944 | 29.5717 | 708 |
| Shenshuige | | 103.4088 | 29.5659 | 807 |
| Chadicunxiagou | | 103.3598 | 29.5965 | 928 |
| Heishuicun | | 103.3610 | 29.6021 | 950 |
| Chadicunshanggou | | 103.3181 | 29.5983 | 1113 |
| Longdonghe | | 103.2827 | 29.5820 | 1242 |
| Longdonghezhiliu | | 103.2816 | 29.5785 | 1248 |
| Linggonglizhugou | | 103.2916 | 29.5855 | 1320 |
| Linggonglizhigou | | 103.2908 | 29.5864 | 1323 |
| Changshouqiao | | 103.3505 | 29.5594 | 1560 |
| Longqiaogou | | 103.3523 | 29.5522 | 1602 |
| Shuangshuijin | | 103.3209 | 29.5471 | 2243 |
| Jinding | | 103.3332 | 29.5237 | 2865 |

**Table S2** Functional traits of tadpoles. The letter in brackets indicated the function associated with each trait (F: food acquisition, L: locomotion).

| Functional trait | Measure | Ecological meaning |
| --- | --- | --- |
| Mass (F/L) | Log(*M*+1) | Volume, muscle mass |
| Oral disk shape (F) | OD/BMW | Prey shape and food acquisition |
| Oral disk position (F) | OD/BL | Position of prey in the water |
| Eye position (F) | IO/BMW | Prey detection |
| Spiracle position (F) | SS/BL | Swimming and hydrodynamism |
| Gut length (F) | GL/BL | Ability to digest food |
| Tail shape (L) | TMW/BMW | Hydrodynamism and Endurance |
| Tail position (L) | TAL/BL | Endurance, acceleration, and/or  maneuverability |
| Tail throttling (L) | TMH/BMH | Propulsion and/or maneuverability |
| Body section shape (L) | BMW/BMH | Position in the water column and hydrodynamism |
| Trunk bending shape (L) | BL/TL | Swimming type (magnitude of lateral bending of the trunk) and endurance |

*M*: mass; TMW: tail muscle width; TAL: tail length; GL: gut length; BL: body length; TMH: tail muscle height; BMH: body maximum height; BMW: body maximum width; TL: total length; OD: oral disk width; SS: distance from tip of snout to opening of spiracle; IO: Interocular distance.

**Table S3** All models in confidence set based on ΔAICc.

|  | Model in confidence set | df | logLik | AICc | ΔAICc | Weight |
| --- | --- | --- | --- | --- | --- | --- |
| richness | Con+Cv+TP+Wd | 6 | 49.15 | -83.97 | 0.00 | 0.30 |
|  | Cv++Wd | 4 | 45.98 | -82.91 | 1.05 | 0.18 |
|  | Cv+TP+Wd | 5 | 47.12 | -82.61 | 1.36 | 0.15 |
|  | (Null) | 2 | 43.32 | -82.34 | 1.63 | 0.13 |
|  | Wd | 3 | 44.38 | -82.13 | 1.83 | 0.12 |
|  | Cv | 3 | 44.30 | -81.99 | 1.97 | 0.11  relative biomass Con+Cv+TP+Wd 6 34.08 -53.83 0.00 0.39  Con+Ele+ Wd 5 32.15 -52.69 1.14 0.22  Con+Cv+Ele+TP+Wd 7 34.84 -52.48 1.35 0.20  Con+ Ele+TP+Wd 6 33.38 -52.43 1.40 0.19 |
| relative biomass | Con+Cv+TP+Wd | 6 | 34.08 | -53.83 | 0.00 | 0.39 |
|  | Con+Ele+Wd | 5 | 32.15 | -52.69 | 1.14 | 0.22 |
|  | Con+Cv+Ele+TP+Wd | 7 | 34.84 | -52.48 | 1.35 | 0.20 |
|  | Con+ Ele+TP+Wd | 6 | 33.38 | -52.43 | 1.40 | 0.19 |

**Table S4** The standard effect size and *P* values calculated by the effects of the extinction of EFE and GFE on the three functional diversity indices. Significant *P* values are in bold.

|  | Number of the extinct entities | SES (extinction of EFE) | SES (extinction of GFE) | *P* value (extinction of EFE) | | *P* value (extinction of GFE) |
| --- | --- | --- | --- | --- | --- | --- |
| FRic | 1 | -2.087 | 0.245 | **0.028** | 0.285 | |
|  | 2 | -2.819 | 0.350 | 0.055 | 0.448 | |
|  | 3 | -3.231 | 0.418 | **0.017** | 0.571 | |
|  | 4 | -3.760 | 0.510 | **0.003** | 0.594 | |
|  | 5 | -4.010 | 0.562 | **0.001** | 0.649 | |
|  | 6 | -4.068 | 0.654 | **0.000** | 0.712 | |
|  | 7 | -4.219 | 0.698 | **0.000** | 0.703 | |
| FOri | 1 | -1.497 | 0.120 | 0.058 | 0.555 | |
|  | 2 | -2.281 | 0.175 | **0.039** | 0.560 | |
|  | 3 | -2.597 | 0.283 | **0.018** | 0.589 | |
|  | 4 | -2.930 | 0.318 | **0.007** | 0.595 | |
|  | 5 | -3.171 | 0.455 | **0.002** | 0.649 | |
|  | 6 | -3.493 | 0.411 | **0.001** | 0.647 | |
|  | 7 | -3.931 | 0.465 | **0.000** | 0.663 | |
| FSpe | 1 | -1.624 | 0.190 | 0.081 | 0.457 | |
|  | 2 | -2.293 | 0.259 | **0.030** | 0.548 | |
|  | 3 | -2.918 | 0.306 | **0.010** | 0.573 | |
|  | 4 | -3.286 | 0.367 | **0.004** | 0.620 | |
|  | 5 | -3.861 | 0.427 | **0.000** | 0.626 | |
|  | 6 | -4.199 | 0.508 | **0.000** | 0.666 | |
|  | 7 | -4.627 | 0.509 | **0.000** | 0.678 | |
